# A metaverse-based virtual reality platform for online certification using Web3

**DOI:** 10.1371/journal.pone.0328620

**Published:** 2025-08-12

**Authors:** Abdul Razzaq, Ahmed B. Altamimi, Wilayat Khan, Mohammad Alsaffar, Fahad F. Alfaisal, Salman Ahmed, Tao Zhang

**Affiliations:** 1 School of Software, Northwestern Polytechnical University, Xian, Shaanxi, People’s Republic of China; 2 Department of Computer Engineering, University of Ha’il, Ha’il, Saudi Arabia; 3 Department of Information and Computer Science, University of Ha’il, Ha’il, Saudi Arabia; 4 Digital Futures Institute, University of Suffolk, Ipswich, United Kingdom; Guangdong University of Petrochemical Technology, CHINA

## Abstract

Context: Metaverse is an emerging technology that synchronizes physical and virtual things. It is used to communicate and simulate the virtual world with the physical world through human actions in real-life scenarios. Combining blockchain and metaverse technologies produces an archetype shift in the educational technology domain regarding online certification, largely due to the impact of synchronizing educational technologies. The combined technology elevates the security measure, ensures transparency, enhances accountability, and reduces costs for the online certification process. Proposed Solution: The suggested solution (MetaEduTech) accelerates the certificate verification process by (i) extenuating the risks of misuse by leveraging decentralized storage of the InterPlanetary File System (IPFS), (ii) securing the certificate, and (iii) providing the metaverse environment for certification. We perform experiments and evaluate the MetaEduTech solution by deploying a blockchain-based smart contract model on Ethereum on the Microsoft Windows platform. Results and Implications: The evaluation results show (i) the efficiency of the query response (5 ms–50 ms), (ii) and the performance of the query execution (CPU utilization between 2%–6%). The findings in this research underscore the effectiveness of the proposed solution with the potential to modernize the certification exam process. The proposed solution and its evaluation can provide insights into how to address the persistent issues surrounding certificate authenticity related to academic verification in a metaverse environment.

## Introduction

Blockchain technology in education is at its early stages with limited adoption in a broader educational and learning context [1]. Although some organizations use it to verify student achievement, there is substantial untapped potential for further exploration on blockchain-based education technologies, i.e., BC for EduTech [2]. Experts believe blockchain can significantly impact the education industry, potentially expanding learning opportunities while challenging the traditional role of educational institutions in certification [3,4]. Currently, the metaverse is a state-of-the-art immersive technology tool that enables the construction of virtual environments. It also supports real-time interaction among users [4]. These interactions create participation in real-world scenarios. Literature reviews depict that using the Metaverse can increase the students’ capabilities [5]. With the demand of educational institutions like Universities, immersive technology is rapidly expanding, specifically for interactive, problem-based teaching. It helps to minimise the distance between students and educators and makes the learning more inclusive, immersive [5]. Also enables time- and location-independent access to instructional resources, particularly through wearable computing and extended reality devices [6].

Specifically, during COVID-19, online learning has shifted its gears from conventional learning to Information technology platforms. These vital and reliable educational technology platform provides a strong and hands-on learning experience to students [7]. Metaverse was on the platform among all digital media, largely recognized and supporting remote learning. These surges are more influenced by academic institutions providing their students a real-time 3D environment for collaboration [2]. The metaverse provides a state-of-the-art platform to access the independent instructional resources, which are mainly accessed through wearable computing and devices. It provides a real-time problem solution and tactical understanding [8] [3].

The online educational institutes are vital for modern and updated education to keep providing them with training to meet market needs. The main concerns are customising the tasks and projects to meet market demands and raising individual skills and capabilities. [9]. The fusion of e-learning and artificial intelligence further amplifies its effectiveness, creating tailored learning experiences for students [10]. Several tools, such as Second Life, have gained recognition for their teaching potential and research opportunities within virtual reality education to foster situational cognition, action learning, and experiential learning within educational institutions [11]. Students may also be able to engage in various social activities by creating and exploring virtual worlds, engaging with virtual characters, and participating in different social activities through platforms such as the Metaverse. Simulating real-world interactions in a three-dimensional environment and facilitating real-time interaction between teachers and students [8].

**Fig 1:**
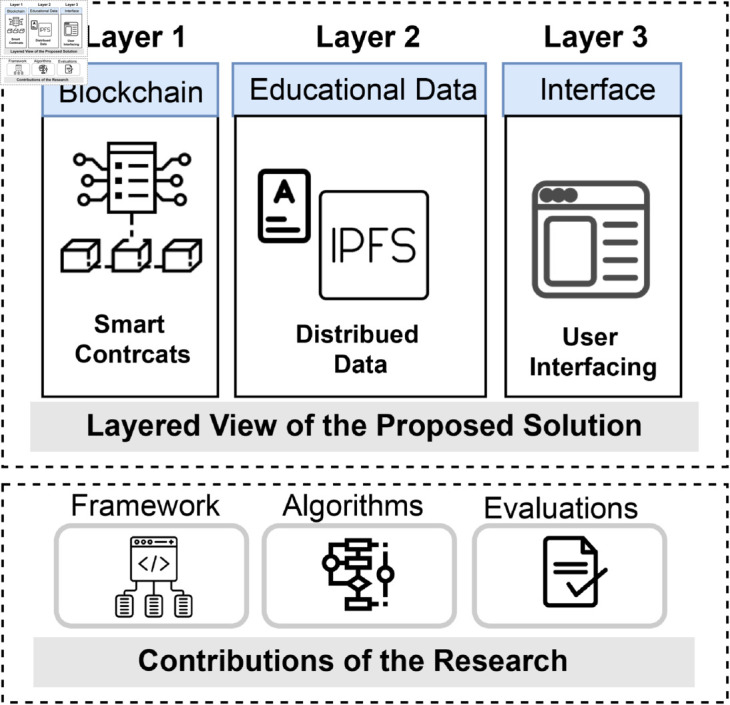
An overview of the proposed solution.

**Proposed solution and contributions:** This paper presents a solution for developing a certification system based on blockchain and metaverse technology. This approach allows users to attend an exam test in the metaverse environment; the certificate will be awarded once the test is passed, and the certificate is saved on decentralized storage and the grade is recorded in the blockchain ledger, as overviewed in Fig 1. Also, for the system to be effective, the integrity and transparency should always be guaranteed. Our approach differs primarily from previous publications because we include extended student records in the form of certifications and grades stored in blockchain ledgers and certification test environments in the metaverse. A key benefit of this research is that it facilitates hands-on analytical and critical thinking experiences within the Metaverse environment. The novelty and contributions of this research are:

- Blockchain-Based Certification in the Metaverse: We introduced a novel blockchain-based approach and model that enables users to take online tests and exams for certification within the metaverse environment.
- Secure Certificate Decentralization: Employing advanced symmetric encryption techniques with dynamic secret keys, we ensure the secure decentralization of certificates upon successful completion of tests and exams.
- Comprehensive Testing and Evaluation: To validate the functionality and assess the effectiveness of our proposed model, we conducted multiple test cases as a case study. These evaluations encompassed key performance metrics, including function execution time, access times, execution costs, and average gas consumption.

*The organization of this paper is structured as follows:* The section on related work reviews the most relevant published research in the context of the proposed solution. The next section, research context and methodology, elaborates on the core concepts for this research and outlines the method to conduct this research. The section on algorithmic implementation of the solution presents the proposed algorithms and explains the utilized technologies, architecture, and the developed model. The section on case study-based evaluation presents a scenario-driven validation of the proposed solution. Finally, the study concludes with key findings and a vision for future work.

## Related work

Firstly, this section discusses blockchain in the educational domain to contextualize blockchain systems and metaverse approaches to explore and develop education systems. Secondly, the section justifies the scope and contribution of the proposed solution, we review and compare the most relevant existing research. This section introduces the concepts and terminology used throughout the paper to justify its value throughout the paper to justify its value.

### Blockchain and educational technologies (BC for EduTech)

Blockchain 1.0, 2.0, and 3.0 phases have been advanced in development. The initial stage, Blockchain 1.0, primarily facilitated straightforward cryptocurrency transactions. In Blockchain 2.0, the focus shifted to real estate and smart contracts, which require specific conditions for registration without intermediaries [12]. The latest stage, Blockchain 3.0, has witnessed diverse applications across sectors like government, education, healthcare, and science. In education, Blockchain is a platform for digital certificates, replacing traditional paper documents. Blockchain technology offers a secure infrastructure for managing certified digital identities governed by programmable smart contracts and recognised certification authorities. The University of Nicosia has adopted blockchain in academics using the Bitcoin blockchain. It helps them to decentralize, secure, and technologically advance verifiable academic certification [13].

Blockchain now holds the key position in education in issuing and verifying digital signatures [14,15]. Its legitimacy is due to its decentralized nature, which provides data integrity and issues a free digital certificate due to its authenticity across platforms [16]. Blockchain also ensures greater credibility and full control over the system’s credentials. It transforms the process from paper-based to a digitized, paperless system and also helps to reduce administrative burden, which eventually leads to transparency and helps to tackle fraud. This eventually leads to increased institutional credibility and the proliferation of qualifications [17]. It also helps institutions streamline their managerial work, including employability support, the acquisition process, and monitoring and tracking progress. This digital revolution in education is reshaping how knowledge is acquired and assimilated, fostering greater student engagement and interaction. Higher education institutions are progressively embracing the integration of e-learning with traditional in-person teaching.

### Immersive technologies: Virtual reality and augmented reality in metaverse

concept of the metaverse, initially introduced in Neal Stephenson’s science fiction work, refers to a three-dimensional virtual world for various activities, including education, we follow the architectural studies [18–20] for explaining the system. Recent definitions [21] describe the metaverse as a technological expression of culture, offering immersive, multi-user online environments where individuals interact socially and economically. However, the educational implications of the metaverse remain unclear, with limited empirical and theoretical studies. Some research explores metaverse learning and teaching platforms driven by technological determinism. From a technological perspective, the Metaverse represents the third wave of the Internet revolution, built on emerging technologies like extended reality (XR), 5G, artificial intelligence (AI), and data processing [22]. Scholars worldwide are rapidly investigating its potential applications in education and the resulting impact on the future of learning.

Within the expansive zone of the Metaverse, one dimension of immersion that garners substantial attention is the utilization of avatars [23]. These digital representations of individuals facilitate interaction and engagement and enable users to navigate this virtual world more effectively. To unlock the Metaverse’s full potential, it is strongly recommended to embrace emerging technologies, and wearable devices, in particular, are poised to enhance the immersive experience, making it more accessible and engaging [24]. While Virtual Reality (VR) and Augmented Reality (AR) have made significant strides in shaping the Metaverse, some scholars have postulated that the future advancement of brain-computer interfaces (BCI) plays a role in expediting Metaverse adoption [25]. BCIs, despite being in the nascent stages of development, hold immense promise, as they can translate brain signals into actionable commands for computers and machinery, opening up exciting possibilities for individuals with cognitive learning impairments and expanding the horizons of accessibility and participation in the Metaverse [26,27].

Noshi and Xu [28] propose a blockchain-based verification system that integrates QR codes for rapid authentication, streamlining the verification process, and reducing fraud. Their system leverages blockchain’s immutability to provide tamper-proof storage of academic credentials. Automating verification through smart contracts enhances security and alleviates the administrative load on educational institutions. Similarly, Abdelmagid *et al*. [29] introduce a permissioned blockchain framework using Hyperledger Fabric, designed to ensure privacy and controlled access while maintaining transparency. Their model restricts participation to authorized entities, which is crucial when handling sensitive academic records. Smart contracts enable automated issuance and validation, fostering a trustworthy environment for credential management. This permissioned approach addresses scalability and privacy concerns more effectively than public blockchains. McGreal [30] explores blockchain’s role in supporting micro-credentials, an emerging trend enabling learners to earn and share smaller units of verified learning achievements. Blockchain’s decentralized and immutable ledger underpins these micro-credentials’ secure management and portability, empowering learners with control over their educational records. While promising, McGreal also highlights challenges related to interoperability and privacy that need to be addressed to maximize blockchain’s educational impact. Cuya and Palaoag [31] focus on blockchain applications in higher education to enhance security, verification, and trust in academic certificates. Their framework combines blockchain with decentralized storage (IPFS) and cryptographic hashing to secure diplomas and transcripts. The system enables employers or institutions to conduct real-time verification, reducing reliance on centralized databases and mitigating the risk of document forgery. They emphasize that permissioned blockchains like Hyperledger Fabric offer performance and privacy benefits suited for educational contexts (Table 1).

**Table 1:** Summary of comparative analysis: Existing vs proposed solution.

Schemes	[6]	[13]	[16]	[17]	Proposed
Encryption ofData	✓	×	×	✓	✓
Storage ofData	IPFS	Blockchain	Blockchain	Blockchain	IPFS
Support ofLarge Data	✓	✓	×	×	✓
*ManagementofDatabase*	Decentralized	Decentralized	Decentralized	Decentralized	Decentralized
Server Attack Resistance	✓	×	×	×	✓
Certificate Process Automation	×	×	×	×	
Flexibility ofMulti Storage	×	×	×	×	✓
No External Authentication	✓	✓	×	×	✓

## Research context and method

This section first provides a comprehensive introduction to the research context, which supports the entire research work. Explaining these fundamental principles equips the reader with the knowledge and understanding required to further investigate the research implications. The section then elaborates the steps of the research method that is employed to conduct this study.

### Research context

**Fig 2:**
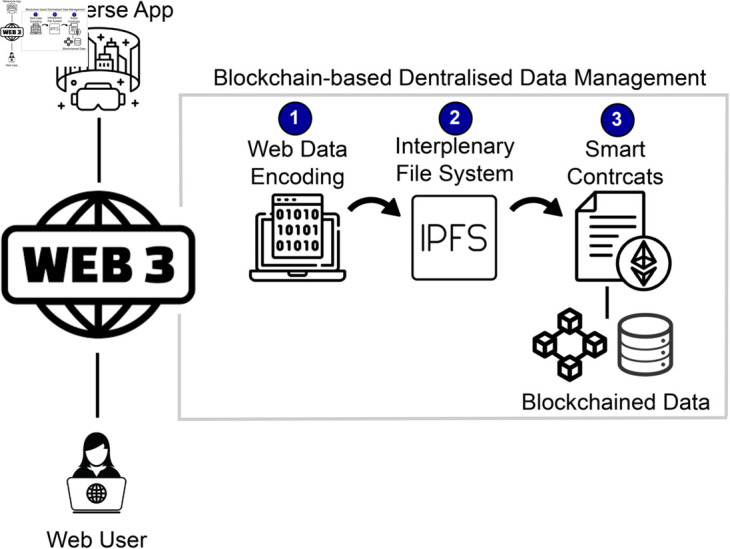
Research context: Web3 and blockchain systems.

This section discusses three sub-phases, including web3, metaverse in education, and secure certification from the education perspective. The solution in this research serves as a robust protection that shifts the technology of the World Wide Web, envisioning the integrity and confidentiality of certificates and personal data as they traverse the blockchain-based metaverse system. Using cutting-edge encryption techniques, we improve the security infrastructure of our system, ensuring that users are protected against unauthorized access or breaches (see Fig 2).

#### The New Age of Web (Web 3.0).

Web3 is a revolutionary paradigm that shifts the technology of the World Wide Web, envisions a new internet era centered around decentralization, blockchain specifics, and cryptographic protection. Through the implementation of decentralized ledger technologies, it endeavors to diminish dependence on centralized authorities, amplifying layers of security, privacy, and trust [32]. The blockchain serves as the basis for a transparent and tamper-proof record-keeping system, linking services with smart contracts to write self-executing agreements and tokenizing to manifest assets digitally.

#### Metaverse and education.

Metaverse and education are at the crossroads of a profound paradigmatic shift in understanding and transferring learning experiences. With the Metaverse, a collective virtual convergence, education seamlessly blends, providing an exciting new environment for teaching and learning. The advancement of technology and the presence of students on internet platforms lead to better engaging experiences for students in understanding education, which is obsolete in the old traditional classroom methods. VR and AR are revolutionising classrooms in the metaverse-based platform world [33]. These technologies provide students with a real-time class environment to help them understand complex concepts effectively. AR and VR are equally important for teachers because they help them create student-centric content focused more on learning preferences and pedagogical needs.

#### Secure certification in education.

In today’s academically competitive world, the credibility of an institution is vital for maintaining student trust and international reputation. The reliability and transparency of academic credentials usually secure it. It is because more learning is now online, and transparency is needed to save teaching from tampering with certification. Digital certifications are rapidly becoming an alternative to traditional paper-based certifications with the help of blockchain technology. These digital certificates are unchangeable ledgers, ensuring the academic records are authentic and protected from unauthorised changes. The challenges arising in blockchain are to create a credible approach, which, possibly in the other way round, leads to deception. Secure certification, an ever-vigilant protector, encompasses its dominion to the restrictions of discomforting piracy and sharp in educational assessments [34].

This research also focused on expanding algorithms intended to streamline data retrieval and authentication processes. These algorithms intentionally enhance the system’s overall functionality and performance. In a dynamic environment where efficiency is paramount, these algorithms are exceptionally tuned to optimize the speed and accuracy of data retrieval, contributing to the unified and speedy operation of the entire system. As shown visually in Fig 2, our research’s algorithmic Solution is essential to the overall structure of our solution. It encompasses a synergy of encryption techniques and data processing algorithms, all working harmoniously to create a secure, efficient, high-performing blockchain-based metaverse system. These carefully designed and implemented algorithms are the backbone of our solution, underpinning its functionality and ensuring the highest standards of security and performance.

### Research method.

**Fig 3:**
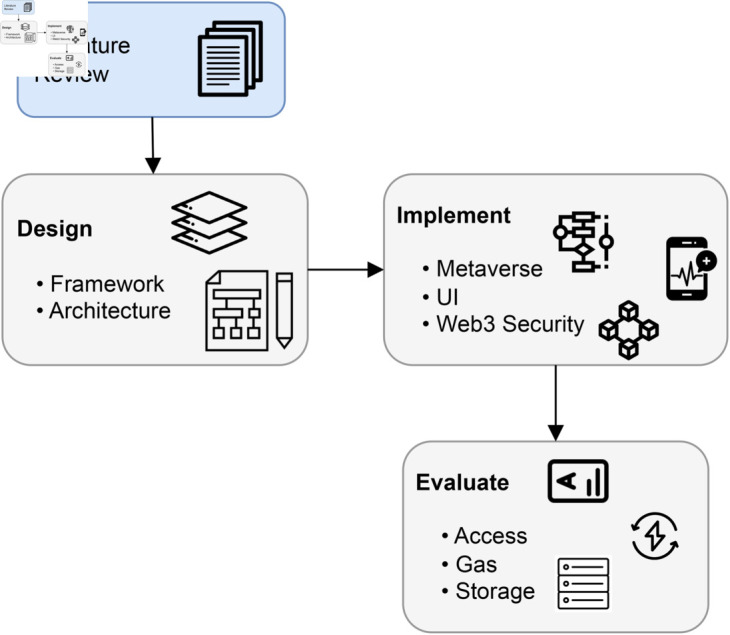
Overviewing steps of the research method.

A comprehensive illustration of the research methodology is thoughtfully presented in Fig 3, meticulously breaking down the entire research process into a series of well-defined steps. This figure serves as a roadmap, guiding the reader through the systematic approach adopted to conduct this research, as elucidated below. A judicious qualitative research methodology was employed to achieve the research objectives effectively. The quantitative methods served as the bedrock for data collection and subsequent analysis, allowing us to amass a wealth of quantitative insights germane to our study, the first step depicted in Fig 3. On the other hand, the qualitative research methods came into play in subsequent phases, marked as Steps II and III in Fig 3.

#### Step I – Literature review and analysis.

Literature Review and Analysis: This initial phase is foundational to our research method. It provides the initial basis upon which our scholarly quest takes root. We are immersed in exploring the principal body of knowledge through the literature at this stage. The aim exceeds a comprehension of the current state-of-the-art; rather, it investigates a comparative journey, roughness established solutions against the background of our proposed innovation. This process reflects the creation of expertise, defining the contours of the research work and serving as our guiding encouragement in subsequent activities.

#### Step II – Architecture and algorithm design.

In this crucial step, we transition from the theoretical foundation placed in Step I to the practical. Here, we design the architecture that underlies our proposed solution. This architectural view is presented in the next section, offering a detailed representation of the structural Solution that supports our research. This step involves the modularized implementation of algorithms essential to our solution. These algorithms are intricately detailed in a next section, illustrating how we plan to translate our theoretical approach into tangible, practical modules.

#### Step III – Solution evaluation.

As the concluding phase within our methodological Solution, this step stands as the support for validating the potency and suitability of our innovative scheme. Here, we focus on our idea as a container of rigorous investigation, methodically searching for its efficiency and evaluating its agreement with the initial problem statement of our research objectives. The details of this evaluative endeavor find particular work in a dedicated next section, where we expand a comprehensive narrative detailing the demonstration of testing, measurement, and validation procedures precisely composed to measure the successful trials of our innovative solution.

This research method unfolds in a structured sequence of steps, starting with a thorough literature review and analysis in Step I, transitioning into the architectural and algorithm design in Step II, and concluding in the rigorous evaluation of our proposed solution in Step III. Each step contributes to the overall progression of the research, guiding us from a conceptual understanding to the tangible implementation and evaluation of our innovative approach.

## Algorithmic implementation of the solution

In this section, algorithms represent the intellectual core logic that powers various facets of our solution. These algorithms have been precisely designed to address and optimize specific architectural components of our innovative approach. They ensure the utmost security and confidentiality of sensitive data within the blockchain-based metaverse system, including certificates and personal information.

### Architectural overview of the MetaEduTech solution

**Fig 4:**
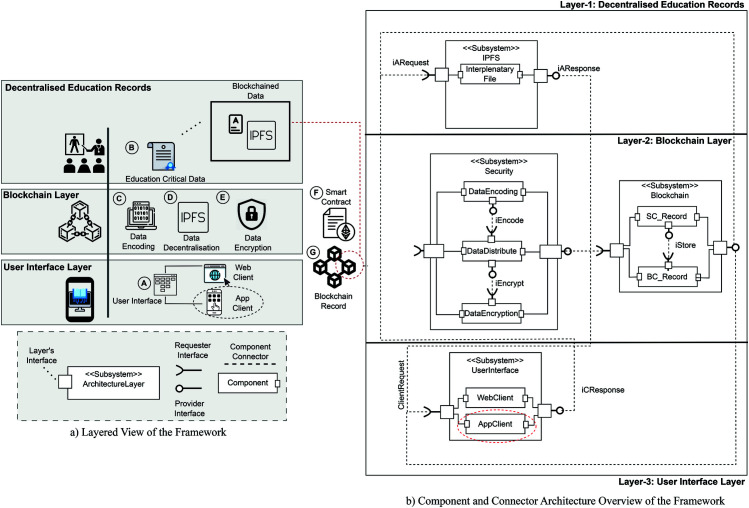
Architectural components view of solution.

Our proposed solution’s architectural view is thoughtfully illustrated in Fig 4, clearly depicting the system’s design. As depicted in Fig 4.

Within this section, our study delves into the comprehensive implementation of algorithms, a core aspect of our research. We meticulously detail and elucidate three principal algorithms, each representing a distinct process delineating the logic governing its implementation. These algorithms are the crux of our research, serving as the backbone of our proposed solution. We have analyzed each algorithm, briefly described its methodology, and are working to demonstrate its role in our research. This is done to demonstrate that the critical analysis of the study offers clarity and transparency in achieving the research objectives. This approach helps us to identify the studies that have explicitly used the integration of VR, which also enables problem-solving in educational institutions. Fig 4 shows how this system has been adapted. In all this process, VR is vital and based on the principle that the system is reliable, innovative, and transparent in issuing certification and demonstrating problem solutions.

**Fig 5:**
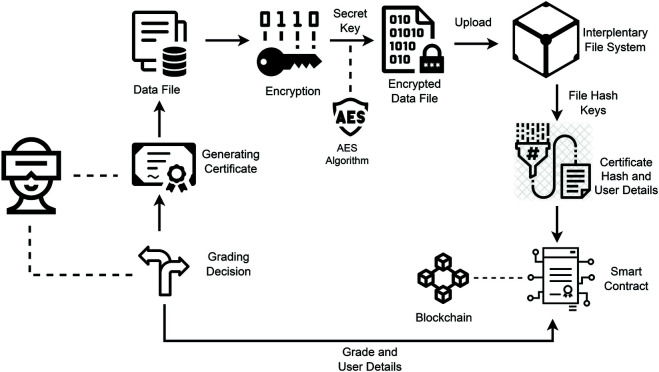
An examination test process for the certification and decentralization.

Fig 5 shows the certificate generated with our proposed solution. This certificate is in PDF format and shares the digital evidence that it is only generated based on the user’s successful test score. The exam/test is created in a user-friendly, dynamic metaverse environment to replicate the immersive learning experience. On completion, the certificate is securely saved in the InterPlanetary File System(IPFS) environment, a decentralized and strong storage solution. We have a factor authentication phase for digital certification to make it secure. In the first phase, the educational institute is encrypted and uploaded to the IPFS System, which assigns each certificate a unique hashable content address. This hashing contains the reference of a certificate file containing the blockchain ledger, along with relevant metadata for transactional data. Other key metadata includes filename, format, description, and file index. This data then integrates with an encrypted file, forming a single artefact ready to be uploaded to IPFS. Pre-tramission, the certificate is coated with a symmetric key algorithm to contain a secure ciphertext version. The ciphertext helps keep data integrity, confidentiality, and resistance to tampering for storage and retrieval. Access to the certificate includes rigorous validation to meet the successful completion of an evaluation process, which makes only an authenticated and eligible user eligible to receive validated credentials. A robust and secure framework is formed by integrating these steps under encryption, decentralised storage, and blockchain verification. This framework helps secure certification, keeping authentication and transparency the main pillars.

### Algorithmic implementations

This section describes the core functionality of the algorithms we are employing in the system. The algorithms are based on operations from personal data and smart contracts to digital certificate handling through encryption. The algorithm maintains a decentralized and transparent system where data can be processed and verified by a relevant, authenticated central authority. Security has been considered at every stage of development to ensure that user data remains private, accurate, and accessible only to authorised individuals. The upcoming sections detail the working algorithm and explain how they contributed to the overall system. We have also demonstrated how these algorithms help maintain a secure and consistent user experience.

#### Algorithm 1 - Generating the metaverse environment.

- *Input(s):* At the core of our blockchain system are several essential input parameters, namely the administration ID, user ID, and the metaverse values. These parameters serve as the foundational building blocks that govern the operations of our blockchain-based metaverse system.
- *Processing:* The Algorithm 1 intricate functionality unfolds as it takes charge of depositing the metaverse virtual reality values. This process is intricately woven with the mapping of tests, a dynamic interaction that involves both the administration, responsible for test creation within the metaverse, and the users, who step into this virtual reality zone to engage with and attempt the tests. The algorithm functions as a dual entity, serving two primary purposes. A key feature of the system is its ability to automatically generate assessments within the metaverse. These tasks place users in interactive virtual settings where they can apply their knowledge and demonstrate practical skills in a realistic, simulation-driven context. Parallel to this functionality, the system manages the user-side interaction with these assessments, ensuring intuitive access and seamless usability through a secure and decentralised interface.
- *Output:* We have embedded a secure framework into our algorithms whose key purpose is to ensure the assessment is secure throughout the process. For this purpose, we are not storing the direct results; rather, they go through an encrypted metaverse system through a verified blockchain. This drastically reduces the risk of tampering, and data are stored more confidentially, with integrity, and preserved over time.

**Algorithm 1. Generating metaverse.**



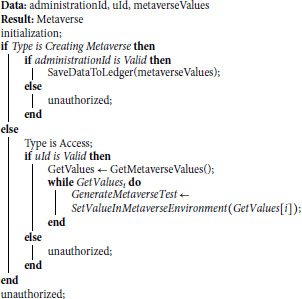



#### Algorithm 2: Decentralised storage with blockchain security.

- *Input(s):* The predefined set of parameters maintains the certification system’s security and trust. These key parameters are user identification and respective performance outcome, blockchain wallet address, and an encrypted issued certificate with a designated key. The process ensures each record is secure and supports overall privacy. This ensures data safety and security throughout the certification process.
- *Processing:* Algorithm 2 is simply the process of issuing a certificate in a decentralised manner. This smart contract will only be active when the user has completed the exam. The process starts with assessment completion, based on which the system generates an encrypted certificate, which is later stored via hashing on the blockchain. The hash contains user-related relevant information, including grading and metadata. Upon failure, a null function will generate a null record instead of recording logs and generating a certificate accordingly. Passing and failure scenarios are covered by this, ensuring no private content is exposed.
- *Output:* The final output of blockchain is based on structured key values stored persistently. The pair consists of the certificate of cryptography stored via hash and the information stored through metadata for the user who has cleared the evaluation. If a user fails to pass the evaluation, a null record will be generated instead of a hash certificate. The key reason behind keeping this uniformity of the data is to ensure that only a valid certificate is stored. This system holds a transparent and secure system to certify the user evaluation records. It only simplifies the verification process and increases the certificate’s strength and overall reliability, which users’ credentials across the blockchain network can trace.

**Algorithm 2. Securing and decentralizing the storage.**



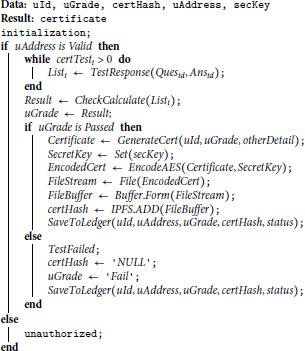



#### System explanation.

The proposed system integrates cryptographic encryption, decentralized storage, and blockchain-backed smart contracts to ensure secure, tamper-proof certificate issuance and access. Initially, a user identified by *uId* submits a set of test responses *R*, which are evaluated by a grading function 𝒢(R). If the user passes, a digital certificate 𝒞 is generated using the user’s identity, grade, time of issuance *t*, and associated metadata ℳ(u). This certificate is encrypted using the AES algorithm ${ℰ}_{AES}^{K}$, where *K* is a symmetric key known only to authorized components. The encrypted certificate is then uploaded to the IPFS, and the resulting content hash ℋ uniquely represents the stored file. Finally, all relevant information—including the user’s ID, blockchain address *uAddress*, grade, encrypted file hash, status *σ*, and timestamp—is sent to a blockchain smart contract 𝒮, which creates an immutable record ${ℬ}_{t}$ on the ledger. This end-to-end mathematical flow ensures the security, integrity, and decentralized accessibility of verifiable digital credentials.

$${ℬ}_{t}=𝒮\left(uId,uAddress,𝒢(R),ℋ\left({ℰ}_{AES}^{K}\left(𝒞\left(uId,𝒢(R),t,ℳ(u)\right)\right)\right),\sigma ,t\right)$$ (1)

## Evaluation: Case study based implementation

This section provides a detailed analysis of the results obtained through implementing the proposed system. It includes a thorough description of the experimental environment to contextualise the evaluation process and support accurate interpretation of the findings. The objective is to present empirical evidence of the system’s operational performance, highlighting its practical applicability, effectiveness, and potential for deployment in real-world educational scenarios. We expound upon the controlled experimental settings established for rigorous evaluations, guaranteeing the results’ reliability and credibility.

### A case study

In this **case study**, we demonstrate the robustness of our solution through the analysis of approximately 50 test transactions attempted by various user accounts in a metaverse environment. Users engage with the system by taking tests presented as visual examples through virtual reality. Upon test completion, the system calculates grades, and for users who pass, the system prompts them to enter a secret key to generate a personalized certificate. This encrypted certificate is decentralized and securely stored on IPFS, granting users access, while the record is simultaneously saved in the blockchain ledger with a certificate hash. In a failed test, a null value replaces the hash. We utilized the Sepolia testnet as our blockchain ledger for transaction storage. Figs 8 and 9 offer a snapshot of a test in the Metaverse and provide insight into the encryption cipher employed for certificate uploads.

**Fig 6:**
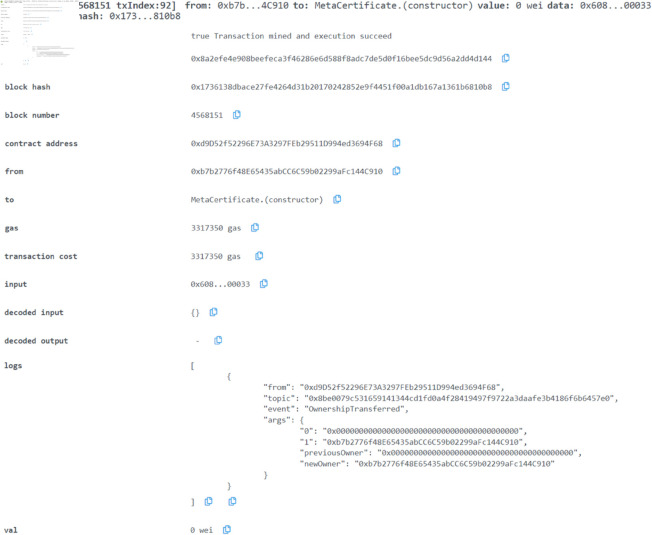
Smart contract deployed and tested.

### Implementation of the solution

**Fig 7:**
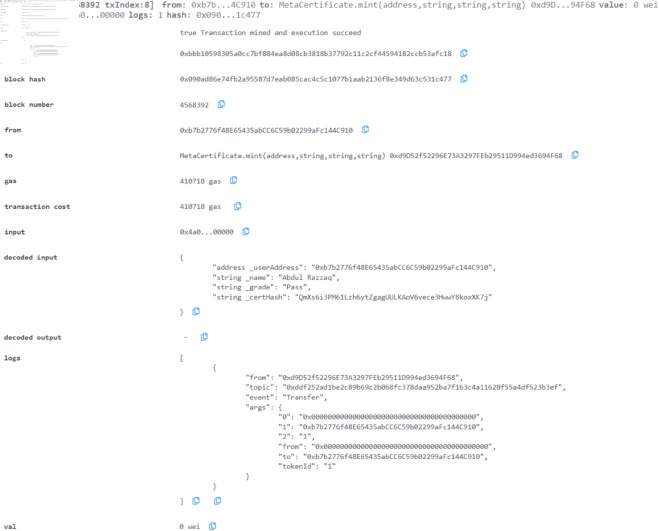
Executed smart contract of metaverse certificate test to save the record.

**Fig 8:**
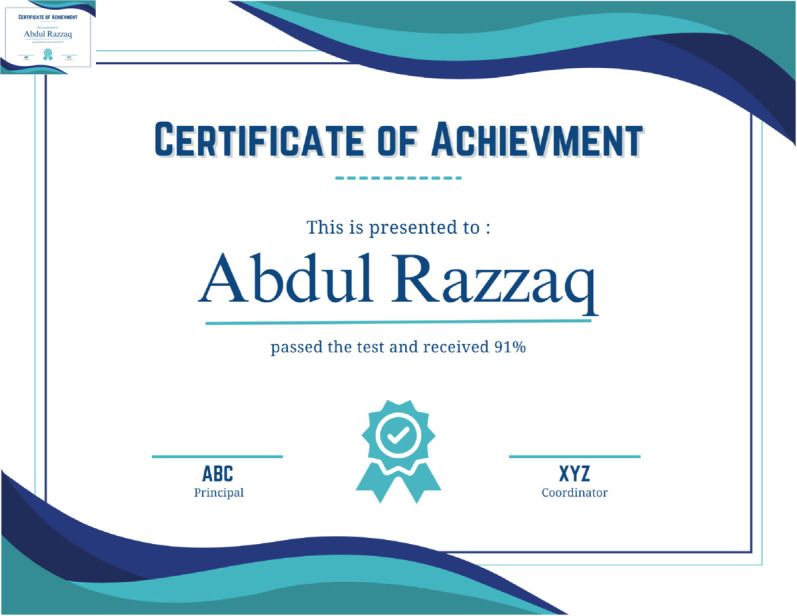
Real certificate generated.

We employed a comprehensive suite of blockchain technologies and metaverse development libraries to operationalize the proposed framework. These components collectively enabled a seamless and secure integration of decentralised certification within an immersive virtual environment. The following subsections outline the technological stack and implementation details:

- **Blockchain Infrastructure:** The core of our Metaverse-based Test System is built upon Ethereum, a widely adopted blockchain platform. Ethereum provides the foundational layer for deploying self-executing smart contracts, which automate certification logic, including issuance, verification, and revocation. These smart contracts record immutable certificate hashes, fostering transparency and data integrity throughout the certification lifecycle.
- **Decentralised Storage:** Successfully issued certificates are encrypted and stored on the IPFS, a distributed storage protocol. IPFS ensures content-addressability and persistence, making it suitable for long-term archival of digital academic records. Each uploaded certificate yields a unique hash, which is then registered on the Ethereum blockchain for verification and retrieval.
- **Non-Fungible Tokens (NFTs):** To represent and manage ownership of metaverse-based digital assets, we utilised Non-Fungible Tokens conforming to the ERC-721 standard. Several Solidity-based libraries were integrated from OpenZeppelin, including:
  1. *ERC721:* Facilitated the creation of unique NFTs that represent individual certificates and assets within the virtual environment. Each token is inherently linked to a user’s blockchain address, ensuring traceable ownership and preventing duplication.
  2. *Ownable:* Provides access control by designating contract ownership to a specific administrative account. This enables secure execution of privileged functions and permits ownership transfer when necessary.
  3. *Counters:* Used to generate sequential and unique token identifiers. It ensures that each NFT minted during the certification process maintains a distinct and tamper-proof identity.
- **Data Security:** We implemented Advanced Encryption Standard (AES) encryption to ensure confidentiality and prevent unauthorized access. This was applied at rest and during transmission, ensuring end-to-end protection of certificate data.
- **Development Stack:**
  1. *Solidity* was used for smart contract development.
  2. *Node.js* facilitated backend logic and algorithm execution.
  3. *Truffle Suite* was employed for contract compilation, deployment, and unit testing within the Ethereum ecosystem.
  4. *Three.js* was used for developing the 3D Metaverse interface, enabling immersive and interactive user experiences.
- **Testing and Deployment:** Initial contract deployment and functional testing were performed using the Remix IDE and the Sepolia Testnet, a Proof-of-Stake blockchain network. This environment allowed free and efficient smart contract deployment, simulating real-world scenarios such as grade recording, certification issuance, and NFT minting for metaverse integration (see Figs 7 and 8).

The integration of these technologies was guided by two key objectives: (i) to simplify deployment for system engineers, and (ii) to uphold rigorous standards for security, reliability, and system interoperability in a virtual reality context. Together, these tools form the technological backbone of our Metaverse-enabled certification system, supporting secure data handling, robust user interactions, and scalable performance.

To enhance the reliability and reproducibility of our findings, we conducted multiple independent trials for both primary operations in our MetaEduTech system: saving grades to the blockchain and generating + decentralizing certificates. Table 2 presents the descriptive statistics of execution times. The mean time to store grades on the blockchain was 10.5 ms (*σ* = 2.1 ms), while the mean time to generate certificates and decentralize IPFS was 27.6 ms (*σ* = 10.8 ms). These relatively low standard deviations indicate stable system behavior across repeated evaluations. The consistency supports the robustness of the proposed architecture under real-world deployment conditions. The statistical consistency of execution times across trials further validates the system’s reliability and readiness for deployment.

**Table 2:** Descriptive statistics for certificate generation and blockchain recording.

Operation	BC: Save Grade	DC: Cert Gen + Decent
1	10	12
2	11	16
3	8	22
4	7	20
5	9	26
6	12	31
7	14	38
8	13	24
9	10	45
10	11	42
11	10	12
12	11	16
13	8	22
14	7	20
15	9	26
16	12	31
17	14	38
18	13	24
19	10	45
20	11	42
**Mean Time (ms)**	**10.5**	**27.6**
**Std Dev (ms)**	**2.12**	**10.82**

Subsequent sections will present a detailed exploration of the system architecture (see Fig 4), modular algorithmic implementation, and evaluation metrics. These components collectively demonstrate the proposed solution’s practicality, efficiency, and educational applicability within decentralised and immersive learning environments.

### Experimental setup for evaluation

We established a purpose-built evaluation environment comprising integrated hardware and software components to assess the proposed system. This setup enabled comprehensive tracking and analysis of system performance across multiple operational phases. On the hardware side, experiments were conducted on a Windows-based platform, the primary host for executing the certificate generation and IPFS upload processes. The system was equipped with an Intel Core i7 processor and 16 GB of RAM, providing sufficient computational resources to support the demands of decentralised certificate processing and storage. A suite of evaluation scripts was developed from the software perspective to automate the assessment procedures. These scripts were primarily written using Node.js, a widely adopted JavaScript runtime environment known for its scalability and asynchronous capabilities. Development and debugging were conducted within the Visual Studio Code IDE, with ReactJS employed to enhance front-end interaction and evaluation logic. Node.js also facilitated seamless integration with the Sepolia testnet, allowing efficient interaction with the deployed smart contracts on the Ethereum blockchain. To ensure precise system performance measurement, the evaluation scripts incorporated specialised JavaScript libraries designed for performance profiling. One such library enabled real-time monitoring of CPU usage during certificate uploads and blockchain interactions, yielding insights into computational efficiency and system resource utilisation. This integrated evaluation framework was instrumental in validating the proposed blockchain-enabled certification system’s reliability, responsiveness, and operational scalability.

### Results of evaluation

This section presents the case study and then discusses the evaluation’s results. We perform criteria-based evaluation by evaluating fuel/gas (cost analysis), exam, and certification generation process (performance).

It is shown in Fig 6 how transactions on the Sepolia Testnet platform are depicted more comprehensively. It shows all the transactions made by the user since they took the test. Fig 7 illustrates all the relevant details relating to the specific transaction block we are interested in. Each block is created according to a particular transaction and contains information about that specific transaction.

As seen in Fig 8, the certificate generated after passing the test is decentralized on IPFS, and the transaction is saved on the blockchain ledger after the certificate has been generated. The template for the certificate has been modified with the name of the system that passed the test written on the certificate by the system who has modified the design and the design.

**Fig 9:**
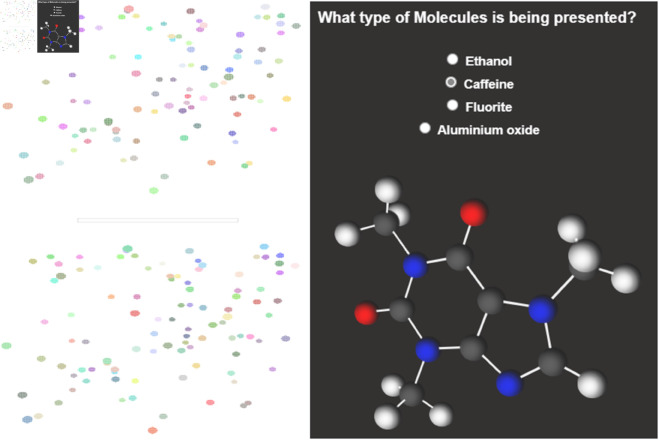
Metaverse visual example as a question for test.

As seen in Fig 9, the certificate generated after passing the test is decentralized on IPFS, and the transaction is saved on the blockchain ledger after the certificate has been generated. The template for the certificate has been modified with the name of the system that passed the test being written on the certificate by the system that has modified the design. Fig 9 shows a detailed representation of the ciphertext used in the certificate. The ciphertext of the generated certificate is stored on IPFS using the AES 256-bit algorithm. The ciphertext is decoded into a certificate when the user wants to download the certificate.

#### Evaluating energy consumption.

**Fig 10:**
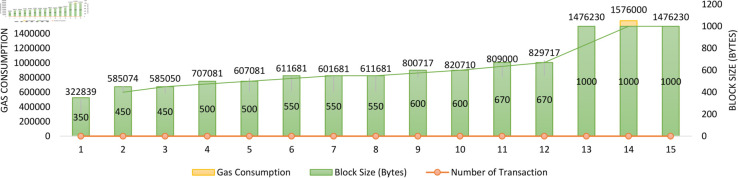
The block size and transaction count are determining factors affecting gas consumption.

We present the energy consumption of the solution (see Fig 10). We performed multiple trials to check the system’s consumption of Ether gas.

The scenario involved a critical operation of saving certificates to the IPFS and the blockchain ledger. Several important performance metrics were examined during this process, and analyzing data. This collective duration, encapsulating data uploading and accessing time, was a key focus of our experiments. In these experiments, we utilized a representative data size, as depicted in Fig 10. The results shed light on the relationship between data size and resource consumption. Specifically, when uploading educational resources exceeding 1000 bytes, the average fuel usage amounted to approximately 1,476,230 gases—likewise, storing data of 350+ bytes consumed an average of roughly 3,22,839 gases. These findings underscore a noteworthy trend: as the size of the data increases, so does the consumption of gasoline. This suggests that resource utilization is directly proportional to data size. Remarkably, even as the data volume increased, there was no noticeable disparity in fuel consumption when certificates were uploaded to IPFS using the proposed method. In essence, this underscores the robustness and scalability of our system, indicating that it consistently maintains efficient fuel utilization regardless of the data size, a crucial aspect of ensuring smooth and reliable performance in the context of blockchain and IPFS integration.

#### Evaluating computational efficiency.

**Fig 11:**
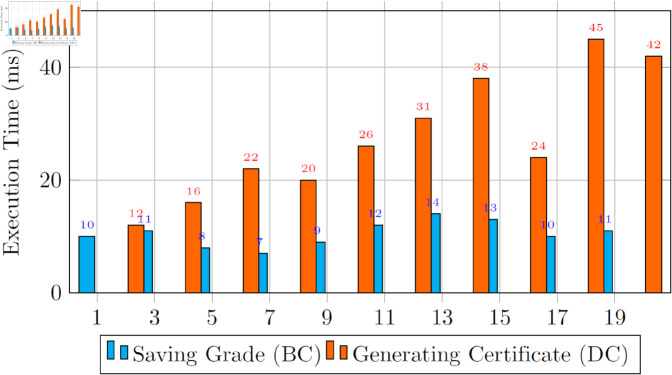
Execution time for saving grades and generating certificates on the blockchain ledger.

**Fig 12:**
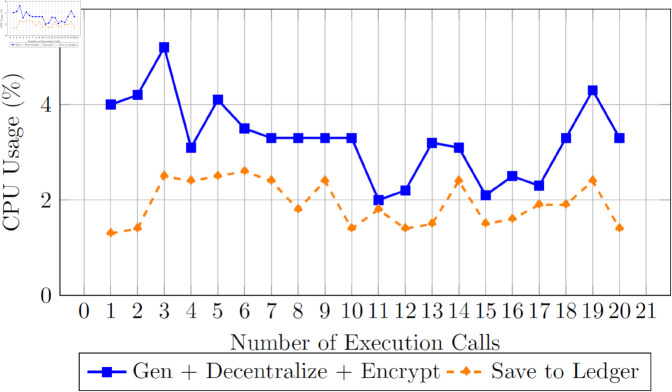
CPU usage during cryptographic and ledger operations over multiple executions.

We now evaluated the developed solution’s computational efficiency with several trials. Fig 11 presents the computational time for transactions to decentralize the certificate and save the record into the blockchain ledger. Fig 12 presents the CPU’s computational time during the trials.

We have implemented a two-pronged approach to ensure the secure storage of certificates on IPFS while maintaining blockchain records. We use the BC label to record information exclusively on the blockchain ledger when users fail their tests. In contrast, the DC label comes into play when certificates are generated upon successful test completion, ensuring decentralization. In evaluating our system’s efficiency, query response time has emerged as an essential metric. This metric provides insights into the system’s data storage and retrieval capacity within the blockchain environment. A series of experiments was meticulously executed to substantiate our approach’s effectiveness. The resulting data, as depicted in Fig 11, showcases our findings. The vertical axis of the figure represents the response time, measured in milliseconds, while the horizontal axis delineates the two distinct execution functions: saving a certificate on IPFS and storing record information on the blockchain via certificate hash.

In Fig 12, a visual representation of CPU usage provides valuable insights into the performance of data access execution. The data access process can be divided into two primary phases: the first phase involves certificate generation and decentralization, followed by the storage of information in the blockchain ledger with a certificate hash when a user successfully passes the test. Conversely, in the event of a test failure, only the grade is stored in the blockchain ledger. Initial stages of execution display relatively longer durations for the first and second numbers. Network latency, resource allocation, and system initialization may cause these delays. Data access efficiency can be negatively impacted by such initial delays, emphasizing the importance of optimizing system performance.

#### Computational efficiency – CPU utilization.

The low CPU usage observed during certificate generation and storage operations, ranging from 2%, indicates minimal processing overhead.

#### Energy efficiency – Gas consumption.

Our gas consumption evaluation (Fig 12) reveals a consistent and linear cost increase about the data size. For example, uploading certificates around 1000 bytes in size required approximately 1,476,230 gas units, while smaller files (350 bytes) averaged around 322,839 gas units. This predictable trend enables reliable forecasting of operational costs, which is essential for scaling the system across thousands of certifications in real-world educational institutions.

#### Performance – Latency.

Performance analysis showed that saving grades to the blockchain averaged 10.5 ms, while generating and decentralizing certificates required approximately 27.6 ms. These latency values support near-real-time feedback and interaction, essential for immersive metaverse-based educational scenarios. Fast response times directly improve user experience and demonstrate the feasibility of integrating MetaEduTech into interactive and time-sensitive learning environments.

## Conclusions and vision for future work

In this publication, we introduce and successfully implement a novel platform that leverages blockchain and metaverse technologies to address prevailing challenges in modernizing existing online test certification systems. Our approach centers on utilizing Ethereum and smart contracts, ensuring the creation of secure, trustworthy, tamper-proof certificates and tests, all within the Sepolia testnet Solution. Within our system, each participating entity possesses unique blockchain addresses, bolstering self-sovereign identity, re-encryption capabilities, and storing requisite data. The algorithms underpinning our solution underwent rigorous cost analysis to assess their efficiency. We executed experimental implementations to thoroughly examine the practicality and logic of the proposed method. This comprehensive examination underscores the system’s capacity to establish secure connections for online test certification within the metaverse. Furthermore, our solution enhances productivity, data provenance, and audit effectiveness, primarily attributed to decentralized data storage, eliminating the necessity for intermediary administrative bodies.

*Needs for future research:* We aim to strengthen the robustness of our evaluations by diversifying data analyses, encompassing a broader number of case studies within the educational metaverse landscape. This expansion enables a more comprehensive evaluation of the proposed Solution’s performance and effectiveness. Such advancements contribute to its overall applicability, aligning it with modern educational practices across various scenarios and contexts.
